# Supramolecular synthons in hydrates and solvates of lamotrigine: a tool for cocrystal design

**DOI:** 10.1107/S2052520624002567

**Published:** 2024-05-10

**Authors:** Gordana Pavlović, Edislav Lekšić, Ernest Meštrović

**Affiliations:** a Department of Applied Chemistry, University of Zagreb Faculty of Textile Technology, Prilaz baruna Filipovića 28a, Zagreb, 10000, Croatia; b Research and Development, PLIVA Croatia Ltd, Prilaz baruna Filipovića 29, Zagreb, 10000, Croatia; c Department of General and Inorganic Chemistry, University of Zagreb Faculty of Chemical Engineering and Technology, Trg Marka Marulića 19, Zagreb, 10000, Croatia; CSIR–National Chemical Laboratory, India

**Keywords:** cocrystals, lamotrigine, solvates, crystal engineering, supramolecular synthons

## Abstract

The molecule of anti-epileptic drug lamotrigine [LAM; 3,5-diamino-6-(2,3-dichlorophenyl)-1,2,4-triazine] is capable of the formation of multicomponent solids. Such an enhanced tendency is related to the diverse functionalities of the LAM chemical groups able to form hydrogen bonds. Two robust synthons are recognized in the supramolecular structure of LAM itself formed via N—H⋯N hydrogen bond: homosynthon, so-called aminopyridine dimer or synthon **1** [*R*_2_^2^(8)] and larger homosynthon **2** [*R*_3_^2^(8)]. The synthetic procedures for a new hydrate and 11 solvates of LAM (in the series: with acetone, ethanol: two polymorphs: form I and form II, 2-propanol, *n*-butanol, *tert*-butanol, *n*-pentanol, benzonitrile, acetonitrile, DMSO and dioxane) were performed. The comparative solid state structural analysis of a new hydrate and 11 solvates of LAM has been undertaken in order to establish robustness of the supramolecular synthons **1** and **2** found in the crystal structure of LAM itself as well as LAM susceptibility to build methodical solid state supramolecular architecture in the given competitive surrounding of potential hydrogen bonds. The aminopyridine dimer homosynthon **1** [*R*_2_^2^(8)] has been switched from *para*-*para* (P-P) topology to *ortho*-*ortho* (O-O) topology in all crystal structures, except in LAM:*n*-pentanol:water solvate where it remains P-P. Homosynthon **2** [*R*_3_^2^(8)] of the LAM crystal structure imitates in the LAM solvates as a heterosynthon by replacing the triazine nitrogen proton acceptor atoms of LAM with the proton acceptors of solvates molecules.

## Abstract 2

Robustness of supramolecular synthons in the lamotrigine solvates and hydrate has been investigated in order to be applied for the lamotrigine cocrystals design.

## 1. Introduction

Understanding the synthon hierarchy and robustness of the systems susceptible to forming multicomponent solids presents a tool for supramolecular design of materials with desired properties (Almarsson *et al.*, 2012 ▸; Trask, 2007 ▸). Solid state chemistry of multicomponent solids has been the focus of pharmaceutical chemistry and industry over the last several decades due to the appearances of various phenomena in such multicomponent solids such as polymorphism (Bernstein, 2007 ▸), hydrates (Morris, 1999 ▸; Khankari & Grant, 1995 ▸), solvates (Griesser, 2006 ▸) or cocrystals, especially API (active pharmaceutical ingredient) cocrystals (Duggirala *et al.*, 2016 ▸). Recently, Bolla with coauthors (Bolla *et al.*, 2022 ▸) published one of the most comprehensive overviews of preparative techniques, design, methodology and crystal engineering of pharmaceutical cocrystals. Such multicomponent solid state forms can modify the physical and chemical properties of pharmaceuticals through the solid state phenomena of mutual molecular recognition and self-assembling via non-covalent interactions. Following the definition of cocrystals given by FDA from February 2018 [US Department of Health and Human Services Food and Drug Administration Center for Drug Evaluation and Research (CDER)] that cocrystals are associated by nonionic and noncovalent bonds/interactions with coformers, one can distinguish among various crystalline solid state forms such as solvates, cocrystals or salts or their subsets such as solvates of cocrystals, solvates of salts or solvates of cocrystals of salts. The classification of cocrystals distinguishes the subset of ionic cocrystals, which are formed from a salt and one or more salts or neutral molecular coformers, from that of molecular cocrystals composed of two or more neutral molecular compounds (Braga *et al.*, 2010 ▸; Haskins *et al.*, 2022 ▸).

Anti-epileptic drug lamotrigine (LAM) [3,5-di­amino-6-(2,3-di­chloro­phenyl)-1,2,4-triazine] shows a tendency to form multicomponent solids such as salts, solvates, cocrystals or their subsets such as solvates or hydrates of LAM salts or LAM cocrystals (Sridhar & Ravikumar, 2011 ▸; Chadha *et al.*, 2011 ▸). The study of multicomponent forms of LAM such as solvates presents a valuable foundation for the study and design of supramolecular synthons for LAM cocrystals from the crystal engineering perspective (Desiraju, 1995 ▸; Aakeröy & Salmon, 2005 ▸; Rodriguez-Hornedo *et al.*, 2007 ▸).

Analysis of the CSD (version 5.45; November 2023) for a structural fragment of the LAM molecule (with any bonds within both rings) identifies 99 entries of any kind of crystalline form of LAM, all with the single-crystal structure and with 3D coordinates deposited (no filters were applied: rerefined structures, new polymorphs, structures at different temperatures were included). The entries were classified manually into 71 salts and solvates of salts, 13 solvates and 15 cocrystals or their solvates (according to requirement that a coformer is in the solid state form at room temperature following the Δp*K*a rule). A detailed list of all multicomponent LAM forms with refcodes is given in Table S1 in supporting information.

In this work, we were particularly focused towards the supramolecular architectures of the crystal structures of 11 solvates (two of them are new polymorphs of LAM ethanol solvate) and a new hydrate polymorph of LAM obtained by single-crystal X-ray diffraction (SCXRD).

The supramolecular architectures of presented LAM multicomponent forms are described based on graph-set analyses of synthons (Etter *et al.*, 1990 ▸; Etter, 1990 ▸).

Comparative structural analysis of previously reported crystal structures of the hydrate derivative and three solvates (two of which are mixtures: ethanol/water and *n*-butanol/water and the third is a 2-propanol derivative with LAM:solvent stoichiometry of 2:2) has been given.

The existence of several stable polymorphic forms contributes to the complexity of the supramolecular architectures of LAM multicomponent crystalline forms.

By applying classifications to crystal forms of active pharmaceutical ingredients, compound LAM *tert*-butanol solvate (1:2) can be regarded as a cocrystal due to the definition of cocrystals since *tert*-butanol as a coformer is in the solid state at 20°C.

**Figure 1:**
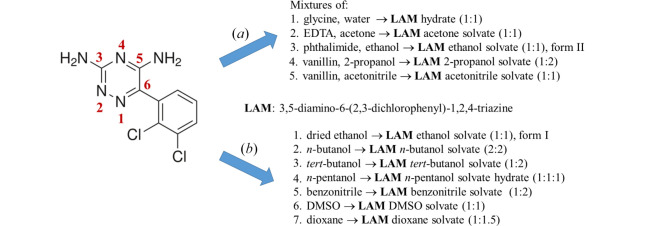
Overview of syntheses of LAM solvates: (*a*) solvates prepared from the mixtures of LAM and potential coformer in solvent; (*b*) solvates prepared from the mixtures of LAM and solvents. For details of synthetic conditions see Section 1 of supporting information.

The majority of chosen solvent molecules contain the oxygen atom functionality (hydrates, alcohols, DMSO, dioxane) acting as a potential proton donor or proton acceptor in the formation of hydrogen bonds, whereas solvates with benzo­nitrile and aceto­nitrile molecules involve the –C≡N functionality as the potential proton acceptor in the formation of hydrogen bonds (Fig. 1 ▸). In attempts to synthesize cocrystals of LAM with selected coformers in appropriate solvents, we obtained LAM solvates instead of cocrystals [Fig. 1 ▸(*a*)]. Therefore, there is a lot of research to be done in the context of the prediction of the stability of certain crystalline forms of LAM under given preparative conditions.

## 2. Materials and methods

### 2.1. Materials

LAM was supplied from in house (PLIVA, Croatia) with purity of 99.9%. Suppliers of solvents and additional substances are listed in supporting information.

### 2.2. Synthesis of the LAM hydrates and solvates

*General observations*. Coformer selection methods in the preparation of LAM cocrystals included synthon probability statistics based on analysis of data in the Cambridge Structural Database (CSD; version 5.45, November 2023). In this work, preparation of 12 LAM multicomponent compounds containing molecules of solvents of crystallization is described. Several solvates of LAM crystallized from the mixtures of LAM and potential coformer in chosen solvent as a result of competitiveness between solvent and coformer molecules for more robust synthon formation with the LAM molecule under given preparative conditions. LAM solvates with ethanol, acetone, water, 2-propanol and aceto­nitrile were isolated in the crystalline form during attempts to synthesize cocrystals of LAM with glycine, EDTA, phthalimide or vanillin (Fig. 1 ▸), and do not necessarily represent the most stable form that can be formed in such systems, which are highly dependent on crystallization conditions.

Other LAM solvates were isolated from the mixtures of LAM with dried ethanol, *n*-butanol, *tert*-butanol, *n*-pentanol, benzo­nitrile, DMSO and dioxane.

Experiments describing procedures for the preparation of single crystals suitable for SCXRD are given in supporting information.

### 2.3. Single-crystal X-ray diffraction

Single-crystal analyses of all compounds were performed on an Oxford Xcalibur Gemini diffractometer equipped with a Sapphire CCD detector and graphite–monochromated Cu *K*α radiation, λ = 1.5418 Å [except for LAM hydrate (1:1): Mo *K*α radiation, λ = 0.71073 Å] at 296 (2) K. All diffraction frames were collected using ω scans. General and crystal data for all compounds are listed in Tables S2–S4 in supporting information. *CrysAlis CCD* and *CrysAlis RED* (Agilent, 2010 ▸) programs were employed for data collection, cell refinement and data reduction (*CrysAlisPro*; Agilent, 2010 ▸). The Lorentz–polarization effect was corrected and the diffraction data have been scaled for absorption effects by the multi-scanning method. The structures were solved by direct methods and refined on *F* ^2^ using the weighted full-matrix least-squares method. *SHELXS97* (Sheldrick, 2015*a* ▸) and *SHELXL2014* (Sheldrick, 2015*b* ▸), integrated in the *WinGX* (v. 1.80.05; Farrugia, 2012 ▸) software package. All non-hydrogen atoms were refined anisotropically. Hydrogen atoms bonded to C*sp* ^2^ and C*sp* ^3^ carbon atoms were placed in geometrically idealized positions with isotropic displacement parameters fixed at 1.2*U*_eq_ [for C*sp* ^2^ carbon atoms] or 1.5*U*_eq_ (for methyl groups) of the atoms to which they were attached and they were constrained to ride on their parent atoms. Hydrogen atoms of the LAM amino groups (bonded to N3 and N5 atoms) as well as the hydroxyl alcohol hydrogen atoms were located in difference Fourier maps as small electron densities at the final stages of the refinement procedures. These H-atom coordinates were refined with isotropic displacement parameters set at 1.2*U*_eq_ of the corresponding nitro­gen or oxygen atoms restraining N—H and O—H distances to 0.86 (2) and 0.82 (2) Å, respectively. In the structure of LAM 2-propanol solvate (1:2), one of the 2-propanol molecules exhibits positional disorder of terminal methyl groups (52:48) with *sp* ^3^–*sp* ^3^ distances restrained by the *DFIX* instruction to 1.51 (1) Å.

Photographs in Fig. S1 show the crystal morphology of the single crystals used for the X-ray diffraction experiment.

The geometries of hydrogen bonds and contacts for all compounds are given in Tables S5–S16.

Molecular geometry calculations (including hydrogen-bonding and non-covalent interactions) were performed using programs *PLATON* (Spek, 2009 ▸) and *PARST* (Nardelli, 1983 ▸, 1995 ▸) integrated in the *WinGX* software system. Molecular visualization with *ORTEP* drawings and packing diagrams were generated using *Mercury* (Macrae *et al.*, 2008 ▸) software.

## 3. Results and discussion

**Figure 2:**
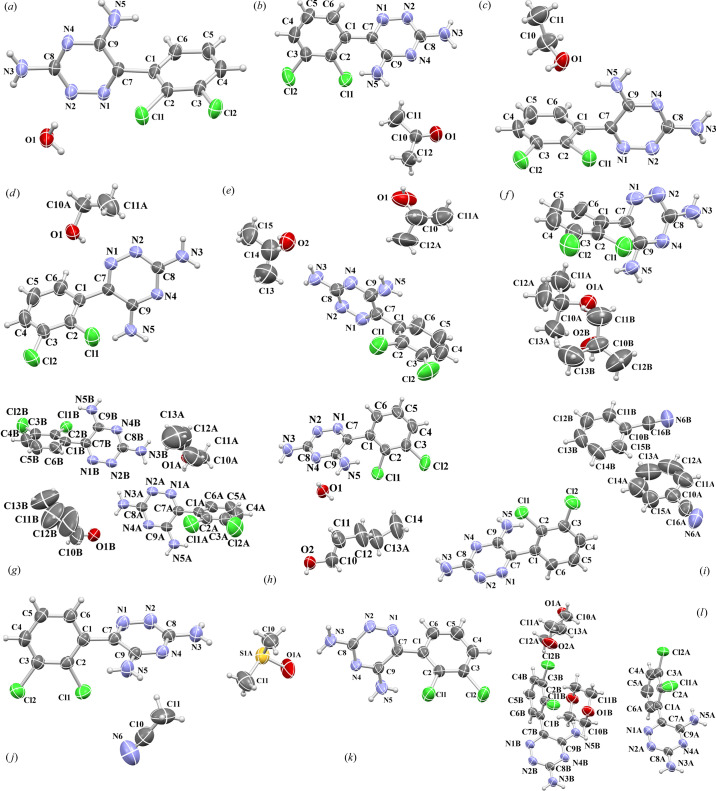
*Mercury*-rendered *ORTEP* drawings of asymmetric units of the molecular structures of LAM solvates reported in this work. Atom-numbering schemes are shown for: (*a*) LAM hydrate (1:1), (*b*) LAM acetone solvate (1:1), (*c*) LAM ethanol solvate (1:1) (form I), (*d*) LAM ethanol solvate (1:1) (form II) (major component denoted as *A* of EtOH disordered molecule is shown), (*e*) LAM 2-propanol solvate (1:2) (only major component denoted as *A* of one 2-PrOH molecule is shown), (*f*) LAM *tert*-butanol solvate (1:2) (two *tert*-butanol molecules are denoted as *A* and *B*), (*g*) LAM *n*-butanol solvate (2:2) (*A* and *B* molecules are denoted), (*h*) LAM *n*-pentanol solvate hydrate (1:1:1) (major component *A* of *n*-pentanol disordered molecule is shown), (*i*) LAM benzonitrile solvate (1:2), (*j*) LAM aceto­nitrile solvate (1:1), (*k*) LAM DMSO solvate (1:1) (major component *A* of disordered DMSO molecule is shown), (*l*) LAM dioxane solvate (1:1.5). Displacement ellipsoids are drawn at the 50% probability level at 296 (2) K.

Molecular structures of LAM hydrate and solvates are shown in Fig. 2 ▸.

A detailed comparative analysis of supramolecular synthons and their relevant geometrical properties in the crystal structures of LAM hydrate and solvates along with the three other solvates (with their CSD refcodes) (Table 1 ▸) is given below.

### 3.1. Analysis of supramolecular synthons

**Figure 3:**
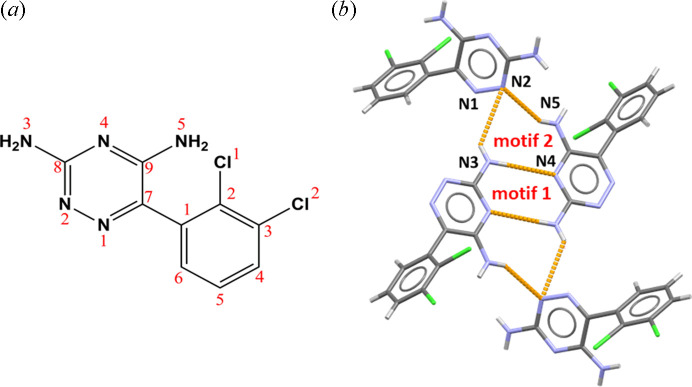
(*a*) Structural diagram of the LAM molecule with the atom-numbering scheme. (*b*) Supramolecular synthons **1** and **2** in the crystal structure of the LAM molecule (CSD refcode: EFEMUX01; usually known as motifs **1** and **2**, respectively) defined *via* the N—H⋯N type of hydrogen bond between the N4 atom of 1,2,4-triazine and the amino N3 atom (synthon **1:** P-P dimerization) and between the N2 atom of 1,2,4-triazine and the amino N3 and N5 atoms (synthon **2**).

Two supramolecular synthons are present in the crystal structure of LAM [Figs. 3 ▸(*b*) and 4 ▸]: synthon **1** 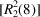 the so-called amino­pyridine centrosymmetrical dimer [involving atoms N3 and N4; *para*-*para* (P-P) dimer] and synthon **2** 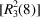 [involving atoms N2, N3 and N5; see Fig. 3 ▸(*b*)]. Synthons **1** and **2** are fused *via* a N3—H5⋯N4 hydrogen bond which acts as a coupling.

Both synthons **1** and **2** form building units in the form synthon **2**–synthon **1**–synthon **2** which are recognized as the more complex supramolecular synthon defined as 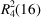 . Synthon 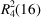 is a robust building block in the crystal engineering of LAM multicomponent solids.

On the basis of the synthon probability statistics, it is well known that the various solvent molecules in the crystal structures of LAM multicomponent solids can participate in the formation of synthon **2** by replacing the N—H⋯N hydrogen bond formed between the amino group and the triazine nitro­gen atom of the LAM molecule with N—H⋯O or N—H⋯N hydrogen bonds formed with proton acceptors of the solvent molecules (Figs. 3 ▸ and 4 ▸).

The investigation is also interesting from the polymorphism (and pseudopolymorphism) viewpoint since some compounds we report here are polymorphs of those previously published (such as the hydrate derivative) (Fig. 1 ▸). We also found two new polymorphic forms of the LAM ethanol (1:1) derivative (assigned by us in this work as forms I and II). So far, the crystal structures of two polymorphic forms discovered at 100 (2) K (Hall *et al.*, 2018 ▸) and 293 (2) K (Évora *et al.*, 2019 ▸) have been published. All four molecular structures exhibit differences in the LAM molecule conformation defining these forms as conformational polymorphs.

Table 2 ▸ and Fig. 5 ▸ present the conformational polymorphism of LAM ethanol (1:1) solvates described so far in the literature with polymorphic forms of LAM ethanol (1:1) solvates discovered by us [see Table 1 ▸: forms I and II, Figs. 2 ▸(*c*) and 2 ▸(*d*)]. The structures assigned CCDC numbers 1483194 and 1826282 are the same compound but determined at 296 K and 100 K, respectively.

#### 3.1.1. Robustness of LAM amino­pyridine synthon **1** in LAM solvates: P-P versus O-O topology

**Figure 4:**
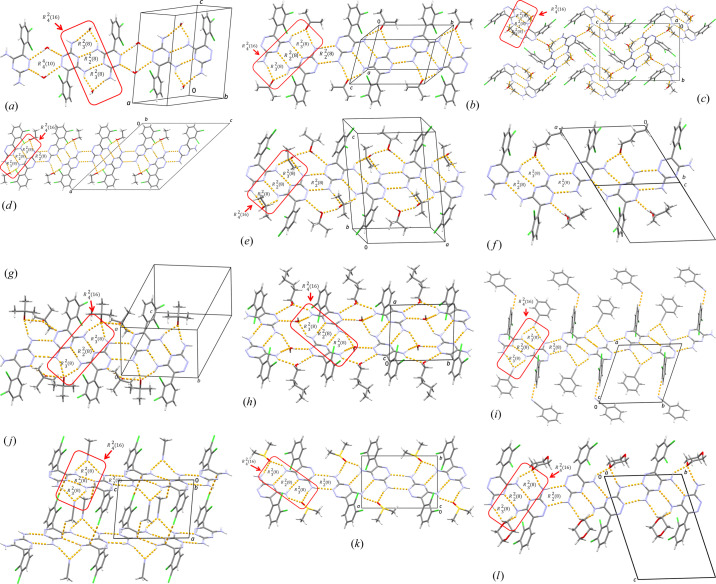
*Mercury*-rendered crystal structures of LAM solvate compounds reported in this work: (*a*) LAM hydrate (1:1), (*b*) LAM acetone solvate (1:1), (*c*) LAM ethanol solvate (1:1) form I, (*d*) LAM ethanol solvate (1:1) form II, (*e*) LAM 2-propanol solvate (1:2), (*f*) LAM *n*-butanol solvate (2:2), (*g*) LAM *tert*-butanol solvate (1:2), (*h*) LAM *n*-pentanol solvate hydrate (1:1:1), (*i*) LAM benzo­nitrile solvate (1:2), (*j*) LAM aceto­nitrile solvate (1:1), (*k*) LAM DMSO solvate (1:1), (*l*) LAM dioxane solvate (1:1.5). For description of each crystal structure, see supporting information.

**Figure 5:**
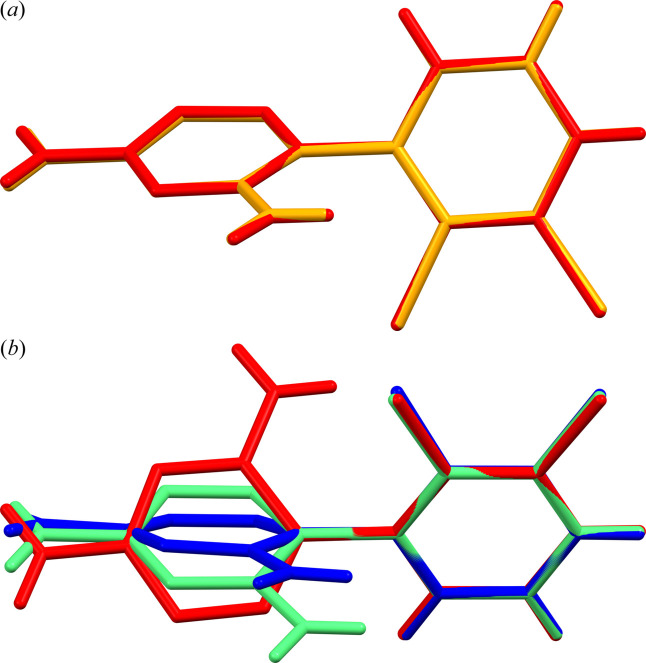
*Mercury*-rendered overlay diagrams exhibiting conformational polymorphism of LAM ethanol (1:1) solvates (see Table 2 ▸). (*a*) Overlay diagram of the same SCXRD molecular structure at room temperature (CCDC number 1483194 – yellow) and at 100 K (CCDC number 1826282 – red). (*b*) Overlay diagram of molecular structures with CCDC numbers 981033 (blue), 148195 (green) and 1826282 (red) exhibiting different spatial orientation of 1,2,4-triazine rings of LAM molecules in three structures [see Figs. 4 ▸(*c*) and 4 ▸(*d*)]; for crystal packing differences in forms I and II, see Section 3.1.3. The overlays have been quantified via RMSD determination.

Aminopyridine synthon **1** interconnecting LAM molecules was found to be present in all analyzed structures converting from P-P topology to O-O topology in all crystal structures, except in LAM *n*-pentanol hydrate where it was preserved as the P-P dimer [Fig. 4 ▸(*h*)]. One polymorphic form of hydrate derivative (CSD refcode XUVLOP; Kubicki & Codding, 2001 ▸) (Table 1 ▸) did not retain synthon **1** due to the water molecules participating in the hydrogen bonding between two LAM molecules which disabled the formation of synthon **1**, probably due to the size of water molecule along with its potential to form hydrogen bonds. On the other hand, in this work, we report the crystal structure of LAM hydrate, the polymorphic form of the XUVLOP structure, where aminopyridine synthon **1** is preserved. Polymorphic LAM hydrate described here was obtained as a coexisting polymorph in an attempt to synthesize the LAM cocrystal with glycine in boiling water (see Section 1 in supporting information). Moreover, we noticed a crystal mixture of transparent and opaque crystals, the latter being a new polymorphic form, and transparent crystals which are confirmed to be a previously published structure (CSD refcode XUVLOP; Kubicki & Codding, 2001 ▸).

#### 3.1.2. Emulation of LAM synthon **2**

The functionalities introduced by selected solvent molecules are capable of imitating synthon **2** 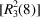 found in the crystal structure of the LAM molecule itself (Fig. 3 ▸) *via* the formation of N—H⋯O or N—H⋯N hydrogen bonds in the case of solvents with oxygen-containing groups or solvents containing –C≡N functionality, respectively.

Structural analysis of LAM solvate structures with solvents in Table 1 ▸ reveals that synthon **2** is not formed only in the structure of the polymorph of LAM hydrate (refcode XUVLOP; Kubicki & Codding, 2001 ▸) (ordinal number 2, Table 1 ▸).

The competition of solvent molecules to hook up to the LAM molecule by hydrogen bonds in the multicomponent LAM solvates containing a water molecule is particularly challenging from the supramolecular point of view [Fig. 4 ▸(*a*): LAM hydrate (1:1) and Fig. 4 ▸(*h*): LAM *n*-pentanol hydrate (1:1:1)].

It is established that emulation of the LAM synthon **2** always happens *via* the N—H⋯O type of hydrogen bond with the water molecule oxygen atom (ordinal numbers 6, 10 and 12 in Table 1 ▸), probably due to the size and hydrogen bond capability of the water molecule.

Condensed synthon 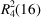 was not found in the crystal structures of water (XUVLOP; Kubicki & Codding, 2001 ▸) and *n*-butanol LAM solvates [Nos. 2 and 9, respectively, Table 1 ▸; Fig. 4 ▸(*f*): LAM *n*-butanol solvate (2:2)].

#### Supramolecular relationship of amino­pyridine synthon **1** 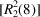 and topologically identical synthon B

3.1.3.

**Figure 6:**
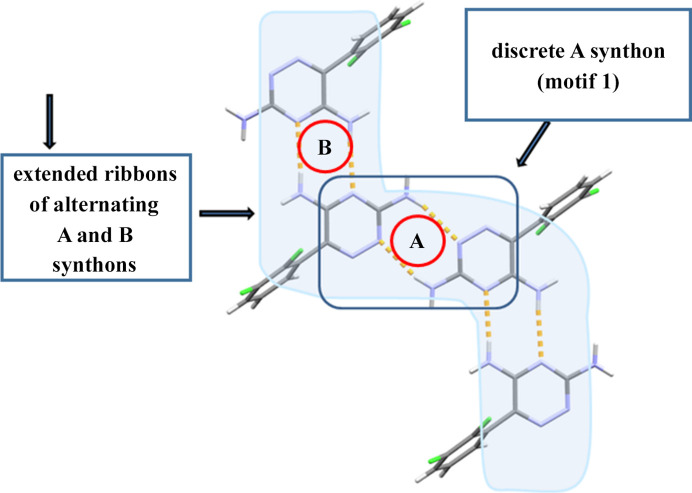
Possible supramolecular assembling of synthon **1** in the crystal structures of hydrate and solvates with the LAM molecule. Assembling of synthon **1** (‘aminopyridine’ synthon) into discrete dimers is more rare than assembling into extended zigzag chains alternating with the synthon denoted as **B** of the same graph-set designation, but of different topology (see Fig. 4 ▸ and Table 1 ▸ for the frequency of appearance in solvates and hydrates of LAM and crystal structure descriptions in supporting information).

The possible supramolecular arrangements of synthon **1** in the described structures are schematically shown in Fig. 6 ▸ showing the assembling of synthon **1** (here, in Fig. 6 ▸ assigned as synthon A also) into discrete dimers or into extended chains with the heterosynthon of the same topology 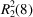 (synthon B) in the alternating AB manner. The extended chains are a more frequent synthon. From Table 1 ▸ it can be seen that discrete dimers are found in four structures: hydrate derivative, polymorph I of ethanol derivative, ethanol/water (WUVLOP; Cheney *et al.*, 2010 ▸) and *n*-butanol/water (OVUNAV; Sridhar & Ravikumar, 2011 ▸) derivatives. In other crystal structures extended chains are present (Table 1 ▸).

Extended planar ribbons of synthons 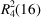 (built up of synthons **1** and **2**) and synthon B [of 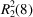 graph-set designation], alternating in the AB fashion, are the most frequent supramolecular architecture of the structures analyzed in this work (Fig. 6 ▸; see also crystal structure descriptions in supporting information and Fig. 4 ▸).

The additional hydrogen bonds are formed with the solvent molecules with the multifunctional donor–acceptor hydrogen bonds capabilities resulting in the additional supramolecular assembling of molecules. This is particularly emphasized for small solvent molecules such as water or simpler alcohols.

## 4. Conclusion

In this article an example of a crystal engineering approach as a potential tool for the design of multicomponent solids of LAM [drug lamotrigine: 3,5-di­amino-6-(2,3-di­chloro­phenyl)-1,2,4-triazine], such as cocrystals, is given. The idea was to describe how the understanding of supramolecular arrangement in the crystal structures of hydrates and solvates can be applied to supramolecular cocrystal design in such a way that the crystal supramolecular topology of the hydrates and solvates is maintained in the crystal structures of cocrystals. If it is established that supramolecular synthons in LAM solvates are robust, this will possibly prevent complete supramolecular synthon rearrangement in LAM cocrystals. In this way, LAM solvates can be used as a design tool for LAM cocrystals.

LAM is an excellent tool that gives deep insight into possible supramolecular arrangements of a model molecule in various supramolecular environments in the crystalline state exhibiting hierarchy of molecular recognition. We previously successfully applied this approach in the preparation of LAM cocrystals (Lekšić *et al.*, 2010 ▸; Lekšić, 2013 ▸).

Supramolecular analysis of a new hydrate and 11 new solvates [with acetone, ethanol with two polymorphs (form I and form II), 2-propanol, *n*-butanol, *tert*-butanol, *n*-pentanol, benzo­nitrile, aceto­nitrile, DMSO and dioxane] of LAM resulted in the observation of robustness of three synthons in the supramolecularly competitive surroundings: synthon **1** of 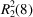 topology, synthon **2** of 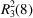 topology and their combination, supramolecular synthon 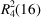 . LAM synthon **1** persists in all solvates except in the hydrate, while synthon **2** of 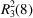 topology is a place for the mutual molecular recognition of LAM with the functional groups of solvent molecules in order to maintain synthon **2** topology.

It is shown that the formation of LAM solvates is highly dependent on crystallization conditions, size of the solvent molecule and its ability to form hydrogen bonds.

Since the LAM molecule itself has a great abundance of hydrogen-bond donors/acceptors as well as molecules of various solvents along with the phenomenon of polymorphism, there is a need for further development and investigations of the crystalline multicomponent forms of the LAM molecule as a fast growing field, particularly from the pharmaceutical point of view.

## Supplementary Material

Crystal structure: contains datablock(s) global, LAM_hydrate, LAM_ethanol_form_I, LAM_ethanol_form_II, LAM_acetone_solvate, LAM_2-propanol_solvate, LAM_n-butanol_solvate, LAM_tert-butanol_solvate, LAM_n-pentanol_solvate, LAM_benzonitrile_solvate, LAM_acetonitrile_solvate, LAM_DMSO_solvate, LAM_dioxane_solvate. DOI: 10.1107/S2052520624002567/aw5084sup1.cif

Structure factors: contains datablock(s) LAM_hydrate. DOI: 10.1107/S2052520624002567/aw5084LAM_hydratesup2.hkl

Structure factors: contains datablock(s) LAM_ethanol_form_I. DOI: 10.1107/S2052520624002567/aw5084LAM_ethanol_form_Isup3.hkl

Structure factors: contains datablock(s) LAM_ethanol_form_II. DOI: 10.1107/S2052520624002567/aw5084LAM_ethanol_form_IIsup4.hkl

Structure factors: contains datablock(s) LAM_acetone_solvate. DOI: 10.1107/S2052520624002567/aw5084LAM_acetone_solvatesup5.hkl

Structure factors: contains datablock(s) LAM_2-propanol_solvate. DOI: 10.1107/S2052520624002567/aw5084LAM_2-propanol_solvatesup6.hkl

Structure factors: contains datablock(s) LAM_n-butanol_solvate. DOI: 10.1107/S2052520624002567/aw5084LAM_n-butanol_solvatesup7.hkl

Structure factors: contains datablock(s) LAM_tert-butanol_solvate. DOI: 10.1107/S2052520624002567/aw5084LAM_tert-butanol_solvatesup8.hkl

Structure factors: contains datablock(s) LAM_n-pentanol_solvate. DOI: 10.1107/S2052520624002567/aw5084LAM_n-pentanol_solvatesup9.hkl

Structure factors: contains datablock(s) LAM_benzonitrile_solvate. DOI: 10.1107/S2052520624002567/aw5084LAM_benzonitrile_solvatesup10.hkl

Structure factors: contains datablock(s) LAM_acetonitrile_solvate. DOI: 10.1107/S2052520624002567/aw5084LAM_acetonitrile_solvatesup11.hkl

Structure factors: contains datablock(s) LAM_DMSO_solvate. DOI: 10.1107/S2052520624002567/aw5084LAM_DMSO_solvatesup12.hkl

Structure factors: contains datablock(s) LAM_dioxane_solvate. DOI: 10.1107/S2052520624002567/aw5084LAM_dioxane_solvatesup13.hkl

Supporting information file. DOI: 10.1107/S2052520624002567/aw5084sup14.pdf

CCDC references: 1483193, 1483194, 1483195, 1483196, 1483197, 1483198, 1483199, 1483200, 1483201, 1483202, 1483203, 2354026

## Figures and Tables

**Table 1:** Comparative analysis of supramolecular synthons in LAM hydrate and solvates Crystal and molecular structures of hydrate (No. 1) and solvates (Nos. 3–5, 7, 9, 11–16) are reported in this work and structures 2, 6, 8 and 10 are identified by their CSD refcode.

No.	LAM hydrate/solvate [with LAM/solvate (or hydrate) stoichiometry]	Presence of synthon **1** †	Presence of synthon **2** †	Supramolecular assembling of synthons (described in Fig. 6 ▸)	Geometry of 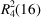 synthon (synthon **2** – synthon **1** – synthon **2** array)	General supramolecular assembling of 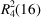 synthon with others†
1	Water (1:1)	Yes	Yes	Discrete dimer	Planar	Extended planar ribbons of synthons 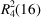 and 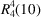 alternate in the AB fashion
2	Water (1:1) (XUVLOP; Kubicki & Codding, 2001 ▸)	No	No	Not formed	Not formed because synthons **1** and **2** are not present	–
3	Acetone (1:1)	Yes	Yes	Extended chains	Planar	Extended planar ribbons of synthons 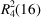 and 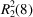 alternate in the AB fashion
4	Ethanol (form I) (1:1)	Yes	Yes	Discrete dimer	Planar	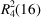 synthon linked into ribbon motifs *via* hydrogen bonds
5	Ethanol (form II) (1:1)	Yes	Yes	Extended chains (zigzag)	Planar	Zigzag ribbons of synthons 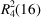 and 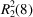 alternate in the AB fashion
6	Ethanol/water (1:1:1) (WUVLOP; Cheney *et al.*, 2010 ▸)	Yes	Yes (*via* H_2_O)	Discrete dimer	Planar	3D array of hydrogen bonds
7	2-Propanol (1:2)	Yes	Yes	Extended chains	Planar	Extended planar ribbons of synthons 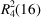 and 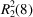 alternate in the AB fashion
8	2-Propanol (2:2) (IJAHOR; Qian *et al.*, 2009 ▸)	Yes	Yes	Extended chains (zigzag)	Planar	Extended non-planar ribbon of alternating 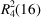 and 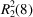 synthons
9	*n*-Butanol (2:2)	Yes	Yes (not centrosymmetric)	Extended chains	Not formed	Extended ribbons of synthons **1** and **2** with another 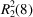 synthon
10	*n*-Butanol/water (1:1:1) (OVUNAV; Sridhar & Ravikumar, 2011 ▸)	Yes	Yes (*via* H_2_O)	Discrete dimer	Planar	Coplanar dimers layered structure
11	*tert*-Butanol (1:2)	Yes	Yes	Extended chains	Not planar	Extended planar ribbons of synthons 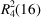 and 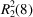 alternate in the AB fashion
12	*n*-Pentanol/water (1:1:1)	Yes	Yes (*via* H_2_O)	Extended chains	Planar	3D hydrogen-bonded network
13	Benzo­nitrile (1:2)	Yes	Yes (*via* N atom of benzonitrile)	Extended chains	Planar	Extended ribbons of alternating 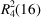 and 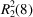 synthons
14	Aceto­nitrile (1:1)	Yes	Yes (*via* N atom of aceto­nitrile)	Extended chains	Aceto­nitrile N atom slightly outside of the synthon **1** plane	Extended non-planar ribbon of alternating 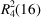 and 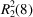 synthons
15	DMSO (1:1)	Yes	Yes	Extended chains	Planar	Extended non-planar ribbon of alternating 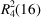 and 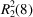 synthons
16	Dioxane (1:1.5)	Yes	Yes	Extended chains	Planar	Extended non-planar ribbon of alternating 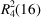 and 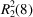 synthons

† LAM synthons **1** and **2** are formed *via* N5—H2N5⋯N4 [amino-pyridine synthon **1** or so-called synthon A (Figs. 3 ▸ and 6 ▸)] and two hydrogen bonds: N3—H1N3⋯N6 and N5—H1N5⋯N6 (LAM synthon **2**, Figs. 3 ▸ and 6 ▸). The centrosymmetric synthon, which does not participate in 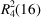 synthon formation, is of the same topology as synthon **1** (Figs. 3 ▸ and 6 ▸), but formed *via* a different hydrogen bond and it is denoted in this work as synthon B (see Section 3.1.3).

**Table 2:** Conformational polymorphs of LAM ethanol solvates

CCDC number	Data collection temperature (K)	Space group and unit-cell parameters (Å, °)	Torsion angle† in the LAM molecule	Topology of homosynthon **1**	Reference
1826282	100	*C*2/*c*, *a* = 21.2458 (15), *b* = 10.2320 (8), *c* = 14.8428 (11), β = 118.808 (4)	−117.6 (7)	O-O (*ortho*-*ortho*)	Hall *et al.* (2018 ▸)
1483194 (form II)	296	*C*2/*c*, *a* = 21.268 (9), *b* = 10.450 (3), *c* = 19.085 (7), β = 136.662 (3)	−117.4 (3)	O-O	This work
981033	293	*C*2/*c*, *a* = 21.300 (2), *b* = 10.471 (1), *c* = 15.024 (1), β = 119.19	117.9 (2)	O-O	Évora *et al.* (2019 ▸)
1483195 (form I)	296	*P*2_1_/*n*, *a* = 7.6894 (3), *b* = 11.3816 (4) *c* = 15.9147 (5), β = 92.710 (3)	92.9 (2)	O-O	This work

† Describes the spatial orientation around the C—C single bond that connects the 2,3-di­chloro­phenyl and 1,2,4-triazine rings of the LAM molecule.
