# Supplementary material for: Supramolecular synthons in hydrates and solvates of lamotrigine: a tool for cocrystal design
Source: Acta Crystallogr B Struct Sci Cryst Eng Mater. 2024 May 10;80(Pt 3):193–200. doi: 10.1107/S2052520624002567 (PMC11157341; doi:10.1107/S2052520624002567)
Supplement: Supplementary file 14 [file b-80-00193-sup14.pdf]

STRUCTURAL SCIENCE
CRYSTAL ENGINEERING
MATERIALS

Volume 80 (2024)

Supporting information for article:

Supramolecular synthons in hydrates and solvates of lamotrigine: a tool for cocrystal design

Gordana Pavlović, Edislav Lekšić and Ernest Meštrović

Table of contents:

1. 1. Materials and syntheses of LAM hydrate and solvates
2. Table S1. Analysis of LAM multicomponent form in CSDB (Ver. 5.45; November 2023)
3. Table S2. General and crystal data for compounds: LAM hydrate (1:1), LAM acetone solvate (1:1), LAM ethanol solvate (1:1), form I and LAM ethanol solvate (1:1), form II
4. Table S3. General and crystal data for compounds: LAM 2-propanol solvate (1:2), LAM *n*-butanol solvate (2:2), LAM *tert*-butanol solvate (1:2) and LAM *n*-pentanol solvate hydrate (1:1:1)
5. Table S4. General and crystal data for compounds: LAM benzonitrile solvate (1:2), LAM acetonitrile solvate (1:1), LAM DMSO solvate (1:1) and LAM dioxane solvate (1:1.5)
6. Figure S1. The photos of single crystals used for diffraction experiments: crystal morphology of LAM hydrate and solvates
7. Figure S2. Supramolecular motifs in the crystal structure of LAM:hydrate (1:1) (refcode: XUVLOP)
8. Figure S3. Supramolecular motifs in the crystal structure of LAM:ethanol:water (1:1:1) (refcode: WUVLOP)
9. Figure S4. Supramolecular motifs in the crystal structure of LAM:2-propanol (2:2) (refcode: IJAHOR)
10. Figure S5. Supramolecular motifs in the crystal structure of LAM: *n*-butanol:water (1:1:1) (refcode: OVUNAV)
11. 2. Crystal structure descriptions
12. Table S5. Geometry of hydrogen bonds and interactions for LAM hydrate (1:1), (Å,°)
13. Table S6. Geometry of hydrogen bonds and interactions for LAM acetone solvate (1:1), (Å,°)
14. Table S7. Geometry of hydrogen bonds and interactions for LAM ethanol solvate (1:1), form I, (Å,°)
15. Table S8. Geometry of hydrogen bonds and interactions for LAM ethanol solvate (1:1), form II, (Å,°)
16. Table S9. Geometry of hydrogen bonds and interactions for LAM 2-propanol solvate (1:2), (Å,°)

17. Table S10. Geometry of hydrogen bonds and interactions for LAM *n*-butanol solvate (2:2), (Å,°)
18. Table S11. Geometry of hydrogen bonds and interactions for LAM *tert*-butanol solvate (1:2), (Å,°)
19. Table S12. Geometry of hydrogen bonds and interactions for LAM *n*-pentanol solvate hydrate (1:1:1), (Å,°)
20. Table S13. Geometry of hydrogen bonds and interactions for LAM benzonitrile solvate (1:2), (Å,°)
21. Table S14. Geometry of hydrogen bonds and interactions LAM acetonitrile solvate (1:1), (Å,°)
22. Table S15. Geometry of hydrogen bonds and interactions LAM DMSO solvate (1:1), (Å,°)
23. Table S16. Geometry of hydrogen bonds and interactions for LAM dioxane solvate (1:1), (Å,°)

1. Materials and syntheses of LAM hydrate and solvates

Vanillin was supplied by Merk, Glycine and EDTA were supplied by Sigma-Aldrich and other organic solvent were supplied by Kemika. No further purification was performed on substances and solvents.

1.1. ***Synthesis of*** **LAM** ***hydrate (1:1)***

A physical mixture containing 100.0 mg (0.4 mmol) of **LAM** and 100.0 mg (1.3 mmol) of glycine is suspended in 10 ml of boiling water. The suspension is allowed to cool and evaporate at room temperature. 7 days after cube like transparent and opaque crystals both suitable for single crystal analysis appeared. Crystal structure of transparent crystal was solved to be known hydrate (CSD refcode XUVLOP), while opaque crystals were found to be a new **LAM** hydrate.

1.2. ***Synthesis of*** **LAM** ***acetone solvate (1:1)***

About 0.5 g (2.0 mmol) of **LAM** and 0.5 g of EDTA (1.7 mmol) was suspended overnight in 5 mL of acetone at 40 °C. Transparent rod like crystals appeared on the top part of crystallization vial.

1.3. ***Synthesis of*** **LAM** ***ethanol solvate (1:1), form I***

100.0 mg (0.4 mmol) of **LAM** is dissolved in 15 mL of dried ethanol in order to remove traces of water. The solution is allowed to evaporate at room temperature. Crystals suitable for SCXRD analysis are obtained after 10 days.

1.4. ***Synthesis of*** **LAM** ***ethanol solvate (1:1), form II***

A physical mixture of 64.0 mg (0.3 mmol) of **LAM** and 36.8 g (0.3 mmol) of phthalimide is suspended in 5ml of warm ethanol. The solution is allowed to cool and evaporate at room temperature. Crystals suitable for SCXRD analysis are obtained after 5 days.

1.5. ***Synthesis of*** **LAM** ***2-propanol solvate (1:2)***

50.0 mg (0.2 mmol) of **LAM** and 29.7 mg (0.2 mmol) of vanillin was dissolved in 5 mL of 2-propanol while heating. The solution is allowed to cool and evaporate at room temperature. Crystals suitable for SCXRD analysis are obtained after 7 days.

1.6. ***Synthesis of*** **LAM** ***n-butanol solvate (2:2)***

50.0 mg (0.2 mmol) of **LAM** was dissolved in 5 mL of *n*-butanol while heating. The solution is allowed to cool. Rod like crystals suitable for SCXRD analysis are obtained after 20 days.

1.7. ***Synthesis of* LAM *tert-butanol solvate (1:2)***

50.0 mg (0.2 mmol) of **LAM** was mixed with 2.0 mg (0.1 mmol) of *tert*-butanol and ground in mortal and paste. Obtained liquid was slowly heated, filtrated and left at room temperature. Thin crystals were taken another day to be analyzed by SCXRD.

1.8. ***Synthesis of* LAM *n-pentanol solvate hydrate (1:1:1)***

50.0 mg (0.2 mmol) of **LAM** was dissolved in 3 mL of *n*-pentanol while heating. The solution was filtered and allowed to evaporate at room temperature. Crystals suitable for SCXRD analysis were obtained after 7 days.

1.9. ***Synthesis of* LAM *benzonitrile solvate (1:2)***

50.0 mg (0.2 mmol) of **LAM** was dissolved in 4 mL of benzonitrile while heating. The solution was filtered. The solution was allowed to cool. Crystals suitable for SCXRD analysis were obtained after 1 day.

1.10. ***Synthesis of* LAM *acetonitrile solvate (1:1)***

50.0 mg (0.2 mmol) of **LAM** and 29.7 mg (0.2 mmol) of vanillin was dissolved in 5 mL acetonitrile while heating. The solution was allowed to evaporate at room temperature. Crystals suitable for SCXRD analysis were obtained after 7 days.

1.11. ***Synthesis of* LAM *DMSO solvate (1:1)***

40.0 mg (0.2 mmol) **LAM** was dissolved in 1.5 mL of DMSO. The solution was allowed to evaporate at room temperature. Crystals suitable for SCXRD analysis were obtained after 7 days.

1.12. ***Synthesis of* LAM *dioxane solvate (1:1.5)***

50.0 mg (0.2 mmol) of **LAM** was dissolved in 6 mL of dioxane. The solution was allowed to evaporate at room temperature. Crystals suitable for SCXRD analysis were obtained after 15 days.

Table S1. Analysis of **LAM** multicomponent form in CSDB (Ver. 5.45; November 2023)

**SALTS**	**SOLVATES (WITH SOLVENT) 11**	**COCRYSTALS (WITH COFORMER) 17**
AYAXOP	FISNED(EtOH)	FISNIH (theofiline)
AYAXUV	GEVYOY (EtOH)	HUQVIA (cinamacid)
FOXLUA	IJAHOR (i-PrOH)	LIGQID (acetamide)
FOXLUA02	KADPAG (MeOH)	QIQHOP (4-methlybenzoate, DMF)
FUHVOU	OVUMUO (β-picoline)	QUHHUZ (tetrahidroizoindole)
FUHVOU02	OVUNAV (ButOH)	SAXHIK
FOXMAH	WUVLIJ(MeOH)	SAXHIK01
FOXMEL	WUVLIJ01(MeOH)	SAXHOQ
GAVLEV	WUVLOP (EtOH/H_2O)	SAXHUW
IWIZAQ	XUVLOP(H_2O)	SAXJAE
KUZMUO	YERTAR (DMF)	VECVEH
LEDTAT		WUVKEE
LIBTUN		WUVKEE01
LIBXUR		WUVKEE02
LIBYAY		WUVKII
NECDAE		XOPVEG (phtalimide)
NESBAQ		YUYQAM (vanillin)
NESBEU		
NESBIY		
NESBOE		
ODUKUW		
OVUMEY		
OVUMIC		
OVUMOI		
PEZKAI		
PEZKEM		
PEZKIQ		
PEZKIQ1		
QIQHIJ		
QIQJAD		
ROJKOS		
ROJKUY		
ROSYIK		
SAVWAQ		
SAVWEU		
SEGGES		
SEGGIV		

SEGGOC		
SEGHAP		
SEGHIX		
SEGHOD		
SEGHUJ		
SEGJAR		
SEGJEV		
SEGJIZ		
SEGJOF		
SEGJUL		
SEGKAS		
VECTOP		
VECTUV		
VECVAD		
WOKXIF		
WOKXOL		
WOKXUR		
WUVKOO		
WUVK001		
WUVKUU		
WUVLAB		
WUVLEF		
WUVLEF01		
YEMZAV		
YEMZAV01		
YEMZID		
YEMZOJ		
YEXFUD		
YUCRAQ		
YUCREU		
YUYQEQ		
ZIJZEB		
ZIJZIF		
ZIJZOL		

Table S2. General and crystal data for compounds: **LAM** hydrate (1:1), **LAM** acetone solvate (1:1), **LAM** ethanol solvate (1:1), form I and **LAM** ethanol solvate (1:1), form II

Compound	**LAM hydrate**	**LAM acetone**	**LAM ethanol (I)**	**LAM ethanol (II)**
Chemical formula	$C_9H_9Cl_2N_5O$	$C_{12}H_{13}Cl_2N_5O$	$C_{11}H_{13}Cl_2N_5O$	$C_{11}H_{13}Cl_2N_5O$
Stoichiometry	1:1	1:1	1:1	1:1
M_r	274.11	314.17	302.16	302.16
Crystal system	monoclinic	triclinic	monoclinic	monoclinic
Crystal dimensions / mm^3	0.20×0.10×0.10	0.6×0.20×0.20	0.30×0.30×0.35	0.50×0.50×0.30
Space group	$P\ 2_1/n$	$P\bar{1}$	$P\ 2_1/n$	$C\ 2/c$
Unit cell parameters:				
a/Å	9.3283(3)	7.1227(6)	7.6894(3)	21.268(9)
b/Å	9.8379(3)	10.6434(10)	11.3816(4)	10.450(3)
c/Å	12.9148(3)	10.9101(13)	15.9147(5)	19.085(7)
α/°		94.031(9)		
β/°	101.405(3)	100.823(9)	92.710(3)	136.662(3)
γ/°		107.105(8)		
V/Å^3	1161.80(6)	469.47(14)	1391.26(9)	2911.1(18)
Z	4	2	4	8
D_c / g cm^{-3}	1.567	1.356	1.443	1.379
μ /mm^{-1}	0.549	0.424	4,211	0.446
F(000)	560	324	624	1248
θ −range / °	4-27	2-24	5-70	3-27

Reflections collected	7485	3812	12626	9035
Reflections unique	2501	2283	2638	3173
Reflections observed [$I > 2\sigma(I)$]	1835	1721	2412	1575
No. of refined parameters	172	193	187	205
$R(F_0)$	0.0576	0.0402	0.0387	0.0413
$R_w(F_0^2)$	0.1093	0.1159	0.1076	0.1050
S	0.998	1.022	1.063	0.813
Max., min. electron density $\Delta\rho_{max}$, $\Delta\rho_{min}$ / e Å^{-3}	–0.274, 0.285	–0.262, 0.164	–0.452, 0.265	–0.309, 0.353

Table S3. General and crystal data for compounds: **LAM** 2-propanol solvate (1:2), **LAM** *n*-butanol solvate (2:2), LAM *tert*-butanol solvate (1:2) and **LAM** *n*-pentanol solvate hydrate (1:1:1)

Compound	**LAM 2-propanol**	**LAM *n*-butanol**	**LAM *tert*-butanol solvate**	**LAM *n*-pentanol hydrate**
Chemical formula	$C_{15}H_{23}Cl_2N_5O_2$	$C_{13}H_{17}Cl_2N_5O$	$C_{15}Cl_6N_2O_4$	$C_{14}H_{21}Cl_2N_5O_2$
Stoichiometry	1:2	2:2	1:2	1:1:1
M_r	376.28	330.21	484.87	362.26
Crystal system	monoclinic	triclinic	triklinski	triclinic
Crystal dimensions / mm^3	0.60×0.38×0.24	0.60×0.38×0.25	0.60×0.50×0.05	0.55×0.55×0.15
Space group	$P\,2_1/n$	$P\bar{1}$	$P\bar{1}$	$P\bar{1}$
Unit cell parameters:				
a /Å	10.5757(10)	10.8596(9)	7.437(3)	7.6284(8)
b /Å	12.8768(19)	11.4487(13)	10.302(3)	8.5181(9)

c/Å	15.0121(17)	15.5823(14)	14.811(3)	14.5190(13)
α/°		98.562(8)	79.70(2)	84.043
β/°		101.242(7)	85.34(3)	75.989(8)
γ/°	95.258(8)	115.425(10)	88.71(3)	85.943(9)
V/Å^3	2035.8(4)	1655.9(3)	1112.8(6)	909.41(16)
Z	4	4	2	2
D_c / g cm^{-3}	1.228	1.325	1.447	1.323
μ /mm^{-1}	3.009	3.582	7.252	3.348
F(000)	792	688	476	380
θ −range / °	5-68	3-68	3-72	3-70
Reflections collected	20445	16409	9500	9619
Reflections unique	3668	6028	4328	3448
Reflections observed [$I > 2\sigma(I)$]	1794	3774	884	2735
No. of refined parameters	256	391	248	238
$R(F_0)$	0.0802	0.0721	0.1293	0.0624
$R_w(F_0^2)$	0.2719	0.2492	0.3864	0.1887
S	0.973	1.040	0.806	1.114
Max., min. electron density $\Delta\rho_{max}$, $\Delta\rho_{min}$ / e Å^{-3}	–0.334, 0.273	–0.299, 0.388	–0.285, 0.469	–0.315, 0.310

Table S4.General and crystal data for compounds: **LAM** benzonitrile solvate (1:2), **LAM** acetonitrile solvate (1:1), **LAM** DMSO solvate (1:1) and **LAM** dioxane solvate (2:2)

Compound	**LAM benzonitrile**	**LAM acetonitrile**	**LAM DMSO**	**LAM dioxane**

Chemical formula	$C_{23}H_{17}Cl_2N_7$	$C_{10}H_{10}Cl_2N_6$	$C_{15}H_{23}Cl_2N_5O_2$	$C_{24}H_{26}Cl_4N_{10}O_3$
Stoichiometry	1:2	1:1	1:1	1:1.5
M_r	462.34	297.15	334.22	644.35
Crystal system	triclinic	triclinic	monoclinic	triclinic
Space group	$P\bar{1}$	$P\bar{1}$	$P\,2_1/n$	$P\bar{1}$
Crystal dimensions / mm^3	0.55×0.35×0.25	0.40×0.40×0.40	0.30×0.25×0.25	0.50×0.50×0.30
Unit cell parameters:				
a/Å	10.3096(8)	7.9855(4)	10.6626(3)	10.2684(12)
b/Å	10.6257(6)	8.6004(5)	7.3529(2)	10.7665(6)
c/Å	12.0489(11)	10.9109(7)	19.6714(5)	14.9036(12)
α/°	74.280(6)	69.598(6)		71.197(6)
β/°	73.318(7)	82.227(5)	92.096(2)	78.435(8)
γ/°	67.161(6)	82.015(5)		74.957(7)
V/Å^3	1154.46(17)	692.48(7)	1541.23(7)	1493.9(2)
Z	2	2	4	2
D_c / g cm^{-3}	1.330	1.425	1.440	1.432
μ /mm^{-1}	2.732	4.192	5.091	3.986
F(000)	476	304	688	664
θ range/ °	4-68	4-70	5-67	5-70
Reflections collected	10040	5383	7391	14176
Reflections unique	4117	2633	2747	5657
Reflections observed [$I > 2\sigma(I)$]	3095	2430	2135	4048
No. of refined parameters	302	185	220	394
$R(F_0)$	0.0430	0.0584	0.0414	0.0748
$R_w(F_0^2)$	0.1151	0.1723	0.1124	0.2061
S	0.925	1.046	0.956	1.076
Max., min. electron density $\Delta\rho_{max}$, $\Delta\rho_{min}$ / e Å^{-3}	–0.204, 0.190	–0.310, 0.732	–0.267, 0.272	–0.575, 0.086

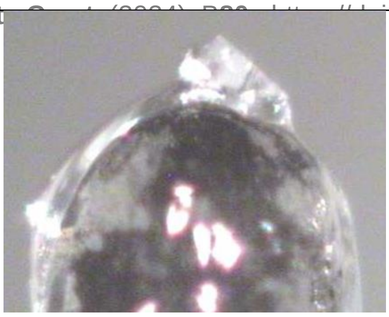	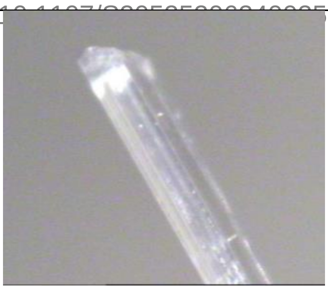	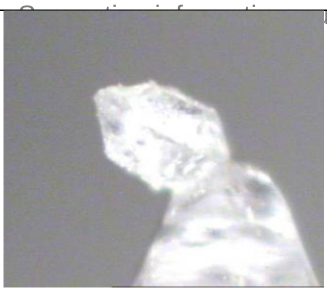
**LAM** hydrate (1:1)	**LAM** acetone solvate (1:1)	**LAM** ethanol solvate (1:1) (form I)
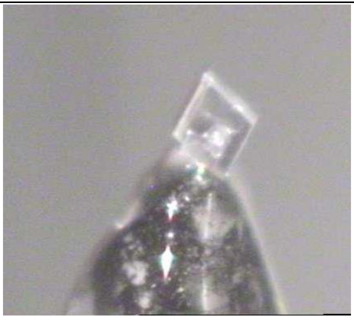	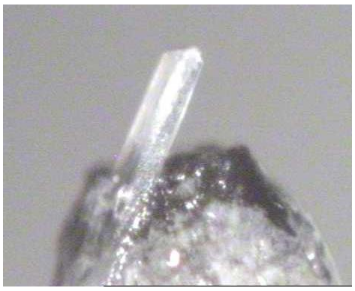	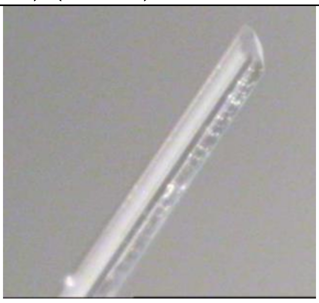
**LAM** ethanol solvate (1:1) (form II)	**LAM** 2-propanol solvate (1:2)	**LAM** *n*-butanol solvate (2:2)
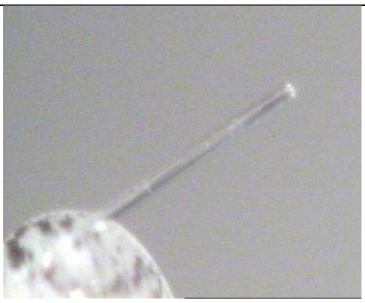	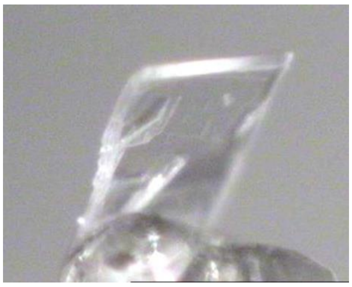	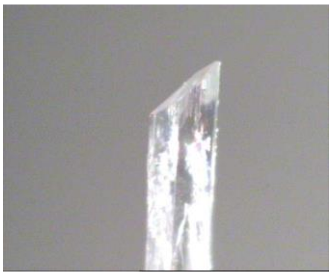
**LAM** *tert*-butanol solvate (1:2)	**LAM** *n*-pentanol hydrate (1:1:1)	**LAM** benzonitrile solvate (1:2)
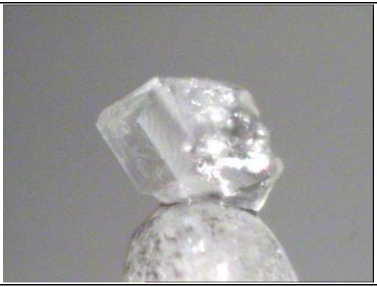	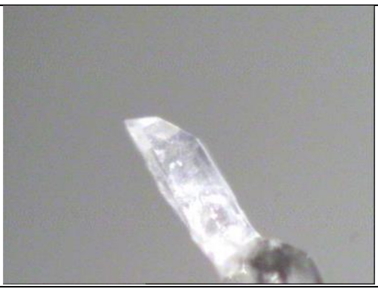	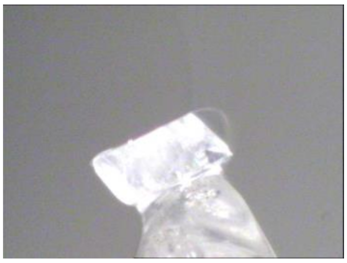
**LAM** acetonitrile solvate (1:1)	**LAM** DMSO solvate (1:1)	**LAM** dioxane solvate (1:1.5)

Figure S1. The photos of single crystals used for diffraction experiments: crystal morphology of **LAM** hydrate and solvates

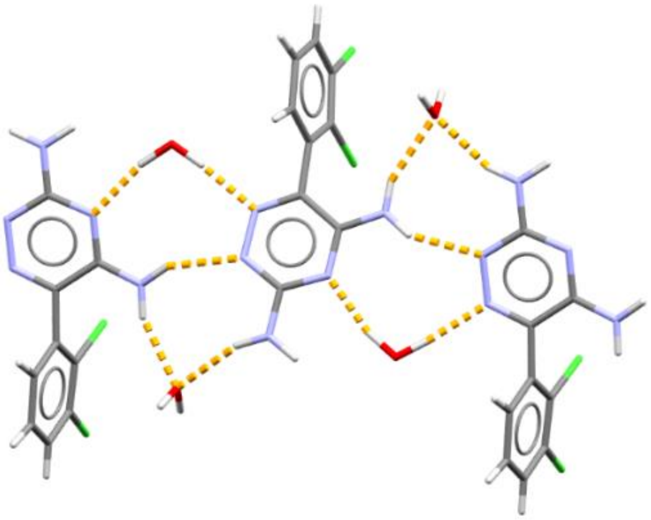

Figure S2. Supramolecular motifs in the crystal structure of **LAM**:hydrate (1:1) (refcode: XUVLOP)

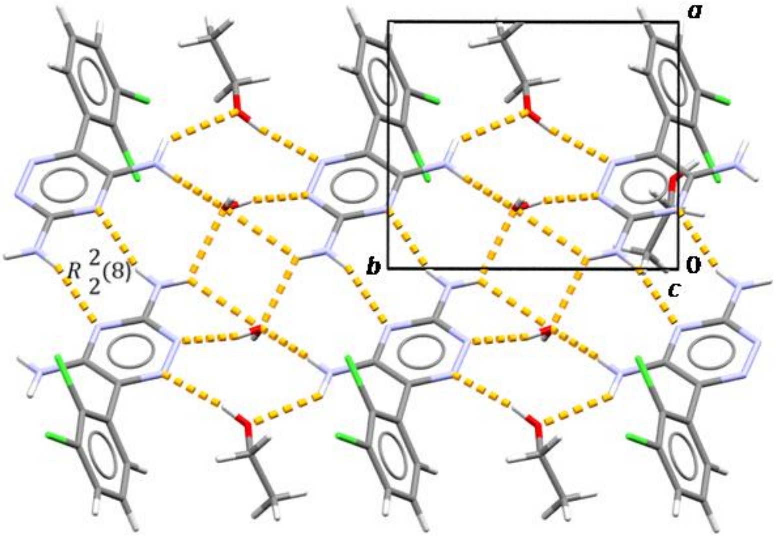

Figure S3. Supramolecular motifs in the crystal structure of **LAM**:ethanol:water (1:1:1) (refcode: WUVLOP)

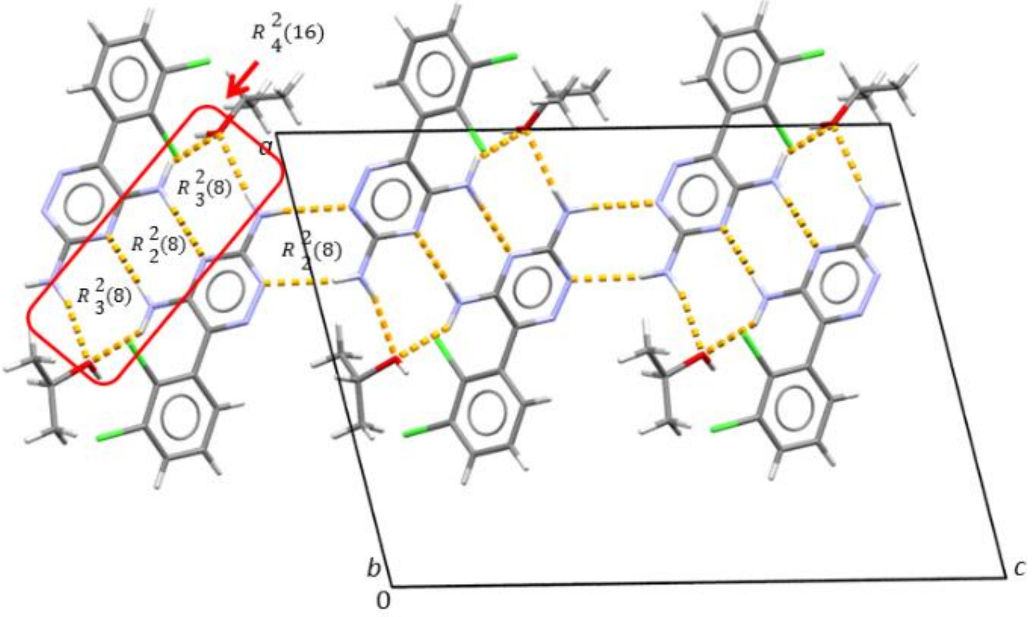

Figure S4. Supramolecular motifs in the crystal structure of **LAM**:2-propanol (2:2) (refcode: IJAHOR)

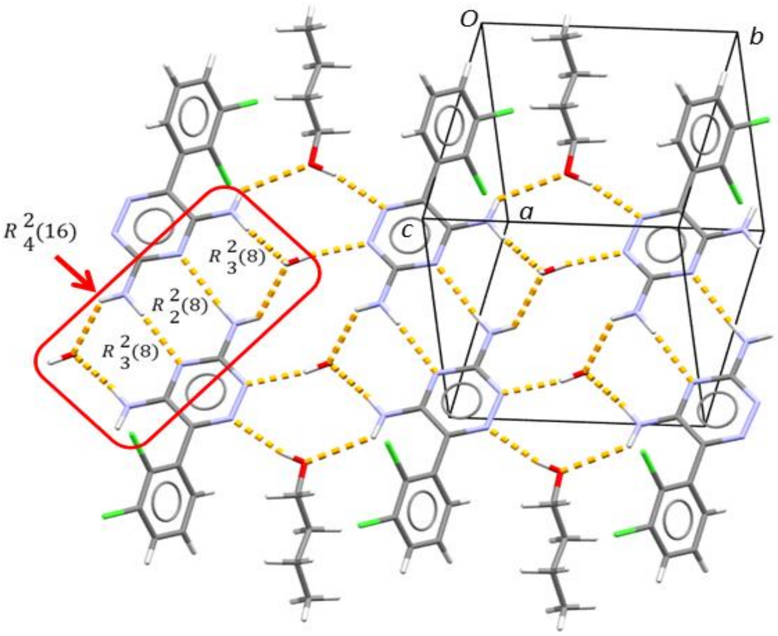

Figure S5. Supramolecular motifs in the crystal structure of **LAM**: *n*-butanol:water (1:1:1) (refcode: OVUNAV)

2. Crystal structures descriptions

2.1. Crystal structure description of LAM hydrate (1:1)

This compound crystallizes in monoclinic space group $P2_1/n$ with one molecule of **LAM** and one water molecule in asymmetric unit. Aminopyridine dimer $R_2^2(8)$ (motif **1** in crystal structure of **LAM** itself) is realized *via* symmetrically equivalent N5–H2N5···N4 hydrogen bond.

These synthons are condensed with $R_3^2(8)$ synthon formed *via* the O1 oxygen atom from water molecule. This condensation produces centrosymmetric $R_4^2(16)$ synthon (combination of motif **1** and **2**). Atom O1 acts as a double proton acceptor forming two hydrogen bonds of the N-H···O type: N5–H1N5···O1 and N3–H1N3···O1. Another centrosymmetric synthon $R_4^4(10)$ is formed by the participation of water molecule as proton donor: *via* O1–H1OA···N1 and O1–H1OB···N2 hydrogen bonds (Table S5). Synthons $R_4^2(16)$ and $R_4^4(10)$ alternates in the AB fashion and form infinite ribbons in one plane. In comparison with known crystal structure of **LAM** monohydrate found in CSD (refcode: XUVLOP) different supramolecular motifs are found. Namely, **LAM** molecule participates in hydrogen bond formation with water molecules which blocks the formation of aminopyridine dimer (motif **1**) between two **LAM** molecules. The motif of infinite ribbons between **LAM** molecules is realized *via* N5–H2N5···N2 hydrogen bond between amino group and the triazine N2 atom.

2.2. Crystal structure description of LAM acetone solvate (1:1)

The compound crystallizes in triclinic $P\bar{1}$ with one molecule of **LAM** and one acetone molecule within asymmetric unit. The aminopyridine **LAM** motif **1**, synthon $R_2^2(8)$ is found. The hydrogen bonds N5–H1N5···O1 and N3–H1N3···O1 between the **LAM** amino groups and the O1 atom from acetone molecule form $R_3^2(8)$ synthon. The condensation of $R_2^2(8)$ and $R_3^2(8)$ synthon results in $R_4^2(16)$ synthon formation. Two such synthons are linked *via* centrosymmetrical $R_2^2(8)$ synthons formed by N3–H2N3···N2 hydrogen bond (Table S6). Supramolecular synthons $R_4^2(16)$ and $R_2^2(8)$ alternates into infinite planar ribbons.

2.3. Crystal structure description of LAM ethanol solvate (1:1) (form I)

Form I crystallizes in monoclinic $P\,2_1/n$ group with one **LAM** molecule and one ethanol molecule. Synthon $R_4^2(16)$ is formed by condensation of aminopyridine $R_2^2(8)$ synthon (motif

1 *via* N5–H2N5···N4 hydrogen bond) (Table S7) with two symmetrically equivalent $R_3^2(8)$ synthons formed *via* N5–H1N5···O1 and N3–H1N3···O1 hydrogen bonds (Table S6) between the **LAM** amino groups and the ethanol oxygen atom O1. The O1-H1O1···N1 and N3-H2N3···Cl1 hydrogen bonds link $R_4^2(16)$ synthon into ribbon motifs spreading along *b* and *c* axes.

2.4. Crystal structure description of LAM ethanol solvate (1:1) (form II)

This solvate crystallizes in monoclinic *C* 2/*c* space group with asymmetric units consists of **LAM** molecule and ethanol molecule. The aminopyridine homosynthon $R_2^2(8)$ realized *via* N5–H2N5···N4 hydrogen bond is preserved in the crystal structure of this solvate (Table S8). By condensation of this synthon with $R_3^2(8)$ synthon formed by the oxygen atom O1 from ethanol molecule as double proton acceptor in two hydrogen bonds: N5–H1N5···O1 and N3–H1N3···O1, the synthon $R_4^2(16)$ is shaped. The hydrogen bond N3–H2N3···N2 (Table S7) between amino group of **LAM** molecule and the atom N2 of another molecule shapes another $R_2^2(8)$ synthon which enables infinite *zig-zag* ribbons of the $R_4^2(16)$ and $R_2^2(8)$ synthons in AB alternating fashion along *c* axis.

In the crystal structure of **LAM** ethanol hydrate (1:1:1) (refcode: WUVLOP; Fig.S3) the different supramolecular architecture is obtained. The coplanarity of synthons (*vs*. *zig-zag* fashion of crystal packing in form II) is achieved by formation of $R_4^2(16)$ synthon *via* the water oxygen atom, while the ethanol oxygen atom participates in hydrogen bond formation with the N1 triazine atom from **LAM** molecule.

2.5. Crystal structure description of LAM 2-propanol solvate (1:2)

Asymmetric unit of monoclinic *P* 2_1/*n* space group consists of one **LAM** molecule and two 2-propanol molecule. One of the two 2-propanol molecules exhibits positional disorder (see Exp. Sect.).The known structure of **LAM** 2-propanol solvate (2:2) (refcode: IJAHOR; Fig.S4) is built up of the two crystallographically independent molecules of **LAM** and two 2-propanol molecules). Two 2-propanol molecules have different crystallographic roles in supramolecular architecture. One 2-propanol molecule participates into $R_3^2(8)$ synthon formation *via* N3–H1N3···O2 and N5–H2N5···O2 hydrogen bonds with the oxygen O2 atom acting as double proton acceptor, while another crystallographically independent 2-propanol molecule is

included into the O1–H11O···N1 hydrogen bond formation with the triazine N1 atom of **LAM** molecule
(Table S9). Simultaneously, the oxygen atom O1 of 2-propanol molecule participates as proton acceptor in the O1–H12O···O2 hydrogen bond formation with another 2-propanol molecule. The N5–H1N5···N4 hydrogen bond forms aminopyridine $R_2^2(8)$ synthon which is condensed with $R_3^2(8)$ into $R_4^2(16)$ synthon. These synthons are assembled into infinite supramolecular motif of condensed alternating coplanar ribbons. In the structure with refcode IJAHOR two molecules of **LAM** and two 2-propanol molecules form synthon $R_4^2(16)$ which is not coplanar with the aminopyridine $R_2^2(8)$ synthon.

2.6. Crystal structure description of LAM n-butanol solvate (2:2)

Asymmetric unit of triclinic $P\bar{1}$ space group contains two **LAM** molecules (A and B) and two *n*-butanol molecules (A and B). Aminopyridine dimer described as $R_2^2(8)$ synthon is preserved between two **LAM** crystallographically independent molecules *via* N5B–H5BB···N4A and N5A–H5AA···N4B hydrogen bonds (Table S10).The atom O1B of one of the *n*-butanol molecules is included in three-centric hydrogen bond formation as double proton acceptor and proton donor. Participation as double proton acceptor enables formation of $R_3^2(8)$ heterosynthon, which is not condensed with the aminopyridine synthon into $R_4^2(16)$ supramolecular synthon due to the sterical hindrance of *n*-butanol. Another *n*-butanol molecule is hydrogen bonded with the triazine N1 nitrogen atom *via* O1A–H1AO···N1A hydrogen bond. Therefore, the infinite ribbon-like motif of **LAM** moieties is realized along with aminopyridine dimer *via* N3A–H3AB···N2B and N3B-H3BA···N2A hydrogen bonds of another $R_2^2(8)$ synthon.

The crystal structure of **LAM** *n*-butanol hydrate solvate (1:1:1) is known (refcode: OVUNAV; Fig.S5) with the asymmetric unit consists of one **LAM** molecule, one *n*-butanol molecule and one water molecule. The supramolecular motif $R_4^2(16)$ is found built up of two synthons: aminopyridine $R_2^2(8)$ motif and $R_3^2(8)$ heterosynthon which is formed by the water oxygen atom as proton acceptor, and not by the alcohol oxygen atom like in **LAM** *n*-butanol solvate.

2.7. Crystal structure description of LAM tert-butanol (1:2)

The compound crystallizes in triclinic $P\bar{1}$ group with one **LAM** molecule and two *tert*-butanol molecules (denoted as A and B) within asymmetric unit. Dimerization of **LAM** molecules occurs *via* N5–H1N5···N4 hydrogen bond (Table S11) implicated the formation of aminopyridine $R_2^2(8)$ homosynthon. The supramolecular motif $R_4^2(16)$, which is not planar due to the sterical requirements of bulky three methyl groups at the tertiary carbon atom, is formed by condensation of aminopyridine $R_2^2(8)$ homosynthon with heterosynthon $R_3^2(8)$ established by participation of the *tert*-butanol oxygen atom O1A as double proton acceptor with N2-H2N5 and N3-H2N3 groups (Table S10). Additionally, this atom participates as proton donor into O–H···O hydrogen bond set up between two crystallographically independent *tert*-butanol molecules. The crystal structure is dominated by continuous ribbon of alternating motifs $R_4^2(16)$ and $R_2^2(8)$ synthon realized *via* N3-H1N3···N2 hydrogen bond.

2.8. Crystal structure description of LAM n-pentanol hydrate (1:1:1)

The aminopyridine $R_2^2(8)$ synthon is preserved in the crystal structure of this solvate *via* N3–H2N3···N4 hydrogen bond. The water molecule participates into N5–H2N5···O1 and N3–H1N3···O1 hydrogen bonds formation (Table S12) as double proton acceptor forming $R_3^2(8)$ synthon condensed with the aminopyridine one into $R_4^2(16)$ synthon. Due to the sterically reason, the formation of $R_3^2(8)$ heterosynthon *via* water molecule O1 atom and not along participation of *n*-pentanol O2 atom is expected. The *n*-pentanol oxygen atom O2 participates as proton donor into O2–H1O2···N1 hydrogen bond formation with triazine ring and as proton acceptor into O1–H1O1···O2 hydrogen bond formation with water molecule. The water molecule forms the fourth hydrogen bond: O1–H2O1···N2.

2.9. Crystal structure description of LAM benzonitrile solvate (1:2)

The asymmetric unit of triclinic $P\bar{1}$ group contains one **LAM** molecule and two benzonitrile molecules (denoted as A and B). The aminopyridine $R_2^2(8)$ synthon is preserved in the crystal structure of this solvate *via* N5–H1N5···N4 hydrogen bond (Table S13). The planar centrosymmetric $R_4^2(16)$ dimer is built up of the aminopyridine $R_2^2(8)$ synthon and the $R_3^2(8)$ heterosynthon formed by N3–H1N3···N6B and N5–H2N5···N6B hydrogen bonds. Triazine

rings as well as both benzonitrile phenyl rings are coplanar. Synthon $R_4^2(16)$ alternates with $R_2^2(8)$ synthon formed *via* N3–H2N3···N2 hydrogen bond into infinite 2D chains of rings. Another benzonitrile molecule, denoted as A, participates into formation of weak C4–H4···N6A hydrogen bond between the N6A atom of nitrile group and phenyl –CH group.

2.10.Crystal structure description of LAM acetonitrile solvate (1:1)

The asymmetric unit of triclinic $P\bar{1}$ group contains one **LAM** molecule and one acetonitrile molecule). The N5–H2N5···N4 hydrogen bond forms the aminopyridine $R_2^2(8)$ synthon, while N3–H1N3···N6 and N5–H1N5···N6 hydrogen bonds forms $R_3^2(8)$ heterosynthon (Table S14). Both these synthons are condensed into $R_4^2(16)$ synthon. The atom N6 is not coplanar with the plane of aminopyridine synthon. Two $R_4^2(16)$ synthons are joined *via* centrosymmetric $R_2^2(8)$ synthon formed by the N3–H2N3···N2 hydrogen bond into ribbon-like infinite supramolecular architecture. The layers of non-planar $R_4^2(16)$ and $R_2^2(8)$ synthons are connected *via* C11–H11A···N4 hydrogen bond.

2.11.Crystal structure description of LAM DMSO solvate (1:1)

The asymmetric unit of monoclinic $P2_1/n$ space group is built up of one **LAM** molecule and one DMSO molecule. The DMSO oxygen atom O1 participates as double proton acceptor into formation of $R_3^2(8)$ synthon *via* N3–H3NB···O1A and N5–H1NA···O1A hydrogen bonds (Table S15). The aminopyridine $R_2^2(8)$ homosynthon also exists, formed by the N5–H5NB···N4 hydrogen bond. Both synthons are condensed into planar $R_4^2(16)$ supramolecular motif which alternates in *ab* plane with centrosymmetric $R_2^2(8)$ homosynthon made by N3–H3NA···N2 hydrogen bond into infinite 2D chains of rings. Ribbons are further joined *via* weak C11–H11B···Cℓ2 and C10–H10B···N2 hydrogen bonds.

2.12. Crystal structure description of LAM dioxane solvate (1:1.5)

Asymmetric unit of triclinic $P\overline{1}$ space group is built up of two **LAM** molecules and 1.5 (due to the symmetry element) dioxane molecules denoted as A and B. The aminopyridine $R_2^2(8)$ dimer, built up of two crystallographically independent **LAM** molecules *via* N5A–H25A···N4B and N5B–H15B···N4A hydrogen bonds, is almost planar (Table S16). Dioxane molecules shape two crystallographically independent heterosynthons $R_3^2(8)$. The O1A atom of dioxane molecule participates into two hydrogen bonds formation: N5A–H15A···O1A and N3B–H13B···O1A, while the O1B atom of another dioxane molecule forms N3A–H13A···O1B and N5B–H25B···O1B hydrogen bonds. In that way, $R_4^2(16)$ synthon is formed. The O1B atom is not coplanar with the plane of $R_4^2(16)$ synthon. Another topologically different $R_2^2(8)$ synthon which is not the constitutional fragment of $R_4^2(16)$ synthon formation alternates with $R_4^2(16)$ synthon forming 2D ribbon-like infinite chains.The weak hydrogen bonds C6A–H6A···N2A and C5A–H5A···N2B are present between layers of $R_4^2(16)$ synthons.

Table S5. Geometry of hydrogen bonds and interactions for **LAM** hydrate (1:1), (Å,°)

D–H···A	D–H	H···A	D···A	∠D–H···A	Symmetry code

O1–H10A···N1	0.81(4)	2.26(3)	3.061(3)	170(2)	−*x*+2, −*y*, −*z*+1
O1–H10B···N2	0.82(2)	2.09(2)	2.908(2)	176(2)	
N3–H1N3···O1	0.87(2)	2.36(2)	3.217(3)	169(2)	−*x*+3/2, *y*+1/2, −*z*+3/2
N5–H1N5···O1	0.86(4)	2.218(2)	2.946(2)	143(1)	*x*−1/2, −*y*+1/2, *z*−1/2
N5–H2N5···N4	0.89(3)	2.12(3)	2.984(3)	171(3)	−*x*+1, −*y*+1, *z* +1
C4–H4···Cℓ2	0.93	2.79	3.642(2)	153	−*x*+2, −*y*+1, −*z*

Table S6. Geometry of hydrogen bonds and interactions for **LAM** acetone solvate (1:1), (Å,°)

D–H···A	D–H	H···A	D···A	∠D–H···A	Symmetry code
N3–H1N3···O1	0.86(2)	2.21(2)	3.040(2)	164(1)	−*x*, −*y*, −*z*+1
N3–H2N3···N2	0.86(2)	2.13(2)	2.983(3)	172(1)	−*x* +1, −*y* +1, −*z* +1
N5–H1N5···O1	0.85(2)	2.17 (3)	2.866(3)	138(2)	*x*+1, +*y*, +*z*
N5–H2N5···N4	0.86(2)	2.20 (2)	3.058(2)	170(2)	−*x* +1, −*y*, − *z*+1

Table S7. Geometry of hydrogen bonds and interactions for **LAM** ethanol solvate (1:1), form I,(Å,°)

D–H···A	D–H	H···A	D···A	∠D–H···A	Symmetry code
O1–H1O1···N1	0.82(3)	2.08(3)	2.891(2)	172(2)	−*x*+1/2, +*y*+1/2, −*z*+1/2
N3–H1N3···O1	0.86(2)	2.66(2)	3.500(3)	167(2)	−*x*, −*y* +1, −*z*+1
N3–H2N3···Cℓ1	0.85(2)	2.92(2)	3.668(2)	148(2)	−*x*−1/2, + *y*−1/2, −*z*+1/2
N5–H1N5···O1	0.86(2)	2.05(2)	2.891(3)	148(2)	
N5–H2N5···N4	0.86(2)	2.14(2)	2.993(2)	174(2)	−*x*, −*y* +1, −*z*+1

Table S8. Geometry of hydrogen bonds and interactions for **LAM** ethanol solvate (1:1), form II, (Å,°)

D–H···A	D–H	H···A	D···A	∠D–H···A	Symmetry code
O1–H1O1···N1	0.82(2)	2.04(2)	2.847(3)	171(2)	
N3–H1N3···O1	0.86(2)	2.00(1)	3.032(2)	164(1)	*x*−1/2, −*y*+1/2, +*z*−1/2
N3–H2N3···N2	0.86(4)	2.19(4)	3.021(5)	163(3)	−*x*, +*y*, −*z*+1/2
N5–H1N5···O1	0.85(2)	2.19(3)	2.870(4)	137(3)	−*x*+1/2, *y*−1/2, −*z*+1/2

N5–H2N5···N4	0.85(2)	2.16(2)	3.010(2)	179(1)	–*x*, –*y*, –*z*

Table S9. Geometry of hydrogen bonds and interactions for **LAM** 2-propanol solvate (1:2),(Å,°)

D–H···A	D–H	H···A	D···A	∠D–H···A	Symmetry code
O1–H11O···N1	0.82(7)	2.31(1)	2.969(6)	138(6)	*x*–1/2, –*y*+1/2, +*z*+1/2
O2–H11O···O1	0.83(5)	2.02(4)	2.809(6)	159(4)	–*x*+1/2, +*y*+1/2, –*z*+1/2+1
N3–H1N3···O2	0.86(7)	2.33(7)	3.171(6)	166(6)	
N3–H2N3···N2	0.86(2)	2.11(2)	2.963(5)	176(2)	–*x*+1, –*y*+1, –*z*+1
N5–H1N5···N4	0.86(7)	2.10(8)	2.963(6)	178(6)	–*x*, –*y*+1, –*z*+1
N5–H2N5···O2	0.86(6)	2.25(8)	2.938(6)	137(6)	–*x*, –*y*+1, –*z*+1

Table S10. Geometry of hydrogen bonds and interactions for **LAM** *n*-butanol solvate (2:2),(Å,°)

D–H···A	D–H	H···A	D···A	∠D–H···A	Symmetry code
N5B–H5BB···N4A	0.86	2.16(1)	3.001(6)	168(3)	*x*–1, +*y*, +*z*
N5A–H5AA···N4B	0.86	2.20(1)	3.006(6)	177(4)	*x*+1, +*y*, +*z*
N3A–H3AB···N2B	0.86	2.18(1)	3.039(5)	165(3)	
N3B–H3BA···N2A	0.86	2.13(1)	2.985(5)	174(3)	
O1B-H1BO···O1A	0.82	1.96(1)	2.717(7)	152(3)	–*x*+1, –*y*+1, –*z*+1
O1A-H1A···N1A	0.82	1.98(1)	2.800(7)	177(1)	

Table S11. Geometry of hydrogen bonds and interactions for **LAM** *tert*-butanol solvate (1:2),(Å,°)

D–H···A	D–H	H···A	D···A	∠D–H···A	Symmetry code
O1A-H1AO···O2B	0.82(2)	1.91(3)	2.733(11)	172(15)	–
N3A–H3AB···N2B	0.86	2.20	2.965(12)	149	–*x*+1, –*y*, –*z*
N3A–H3A···O1A	0.86	2.27	3.094(10)	160	–*x*+1, –*y*+1, –*z*
N5–H5A···N4	0.86	2.19	3.025(10)	165	–*x*+1, –*y*+1, –*z*
N5–H5B···O1A	0.86	2.27	2.986(10)	140	–
O2B–H2BO···N1	0.82(2)	2.15(6)	2.935(10)	160(15)	*x*, *y*+1, *z*
N3–H3A···O1A	0.86	2.27	3.094(10)	160	–*x*+1, –*y*+1, –*z*
N3–H3B···N2	0.86	2.20	2.965(12)	149	–*x*+1, –*y*, –*z*
N5–H5A···N4	0.86	2.19	3.025(10)	165	–*x*+1, –*y*+1, –*z*
N5–H5B···O1A	0.86	2.27	2.986(10)	140	–
O1A–H1AO···O2B	0.82(2)	1.91(3)	2.733(11)	172(15)	–

Table S12. Geometry of hydrogen bonds and interactions for **LAM** *n*-pentanol solvate hydrate (1:1:1),(Å,°)

D–H···A	D–H	H···A	D···A	∠D–H···A	Symmetry code
O1─H1O1···O2	0.82(2)	2.07(2)	2.877(3)	168(2)	─*x*+1, ─*y*+1, ─*z*+1
O1─H2O1···N2	0.82(2)	1.96(2)	2.783(3)	173(2)	*x*, +*y* +1, +*z*
O2─H1O2···N1	0.81(4)	2.10(4)	2.911(3)	172(3)	*x*+1, +*y* +1, +*z*
N3─H1N3···O1	0.87(2)	2.19(3)	2.929(3)	144(2)	─*x*, +1, ─*y*, ─*z*+1
N3─H2N3···N4	0.88(3)	2.22(3)	3.097(3)	177(3)	─*x*, +1, ─*y*, ─*z*+1
N5─H1N5···O2	0.86(3)	2.43(3)	3.087(3)	133(3)	*x*─1, ─*y*, ─ *z*+1
N5─H2N5···O1	0.87(4)	2.01(3)	2.866(4)	168(3)	

Table S13. Geometry of hydrogen bonds and interactions for **LAM** benzonitrile solvate (1:2), (Å,°)

D–H···A	D–H	H···A	D···A	∠D–H···A	Symmetry code
N5─H1N5···N4	0.87(1)	2.11(1)	2.980(2)	177(1)	─*x*, ─*y*+1, ─*z*
N3─H1N3···N6B	0.86(2)	2.43(2)	3.275(3)	166(2)	*x*─1, +*y*, +*z*─1
N5─H2N5···N6B	0.85(2)	2.31(3)	3.027(5)	142(2)	─*x*+1, ─*y*+1, ─*z*+1
N3─H2N3···N2	0.85(2)	2.15(3)	2.990(3)	168(2)	─*x*, ─*y*, ─*z*
C4─H4···N6A	0.93	2.54	3.321(4)	142	─*x*+2, ─*y*, ─*z*+1

Table S14. Geometry of hydrogen bonds and interactions **LAM** acetonitrile solvate (1:1),(Å,°)

D–H···A	D–H	H···A	D···A	∠D–H···A	Symmetry code
N5─H2N5···N4	0.86(3)	2.21(3)	3.056(3)	172(3)	─*x*+2, ─*y*+1, ─*z*+1
N3─H1N3···N6	0.85(3)	2.49(4)	3.326(5)	163(3)	*x*+1, +*y*, +*z*
N5─H1N5···N6	0.86(6)	2.35(6)	3.054(7)	139(4)	─*x*+1, ─*y*+1, ─*z*+1
N3─H2N3···N2	0.86(5)	2.19(4)	3.017(3)	162(3)	─*x*+2, ─*y*+1, ─*z*+2
C11─H11A···N4	0.96	2.73	3.339(5)	122	-

Table S15. Geometry of hydrogen bonds and interactions **LAM** DMSO solvate (1:1), (Å,°)

D–H···A	D–H	H···A	D···A	∠D–H···A	Symmetry code
N5–H5NB···N4	0.87(2)	2.15(3)	3.016(3)	174(2)	–*x*+1, –*y*+1, –*z*
N3–H3NB···O1A	0.87(2)	2.14(2)	2.964(9)	167(2)	
N5–H5NA···O1A	0.86(2)	2.04(3)	2.745(0)	139(2)	–*x*+2, –*y*+1, –*z*
N3–H3NA···N2	0.86(1)	2.13(1)	2.982(2)	176(1)	–*x*+1, –*y*+1, –*z*
C10-H10B···N2	0.96	2.70	3.635(4)	167	*x*, +*y*–1, +*z*
C4–H4···Cℓ1	0.93	2.95	3.558(2)	126	*x*, +*y*+1, +*z*

Table S16. Geometry of hydrogen bonds and interactions for **LAM** dioxane solvate (1:1.5), (Å,°)

D–H···A	D–H	H···A	D···A	∠D–H···A	Symmetry code
N5B–H15B···N4A	0.90(4)	2.19(4)	3.047(5)	173(3)	–*x*+1, –*y*+1, –*z*+2
N5A–H25A···N4B	0.86(5)	2.14(5)	2.996(5)	176(3)	–*x*+1, –*y*+1, –*z*+2
N5A–H15A···O1A	0.86(3)	2.17(4)	2.869(4)	138(2)	–*x*+1, –*y*+2, –*z*+1
N3B–H13B···O1A	0.86(4)	2.24(4)	3.072(4)	162(3)	*x*, +*y*–1, +*z*+1
N5B–H25B···O1B	0.86(2)	2.40(4)	2.975(4)	124(2)	
N3A–H13A···O1B	0.86(4)	2.24(4)	3.092(5)	168(3)	–*x*+1, –*y*+1, –*z*+2
C6A–H6A···N2A	0.93(0)	2.70(0)	3.601(5)	163(0)	–*x*+1, –*y*+1, –*z*+2
C5A–H5A···N2B	0.93	2.66	3.339(7)	131	*x*, +*y*+1, +*z*
